# Mutations at the Subunit Interface of Yeast Proliferating Cell Nuclear Antigen Reveal a Versatile Regulatory Domain

**DOI:** 10.1371/journal.pone.0161307

**Published:** 2016-08-18

**Authors:** Miklos Halmai, Orsolya Frittmann, Zoltan Szabo, Andreea Daraba, Vamsi K. Gali, Eva Balint, Ildiko Unk

**Affiliations:** The Institute of Genetics, Biological Research Center, Hungarian Academy of Sciences, Szeged, Hungary; University of South Alabama Mitchell Cancer Institute, UNITED STATES

## Abstract

Proliferating cell nuclear antigen (PCNA) plays a key role in many cellular processes and due to that it interacts with a plethora of proteins. The main interacting surfaces of *Saccharomyces cerevisiae* PCNA have been mapped to the interdomain connecting loop and to the carboxy-terminal domain. Here we report that the subunit interface of yeast PCNA also has regulatory roles in the function of several DNA damage response pathways. Using site-directed mutagenesis we engineered mutations at both sides of the interface and investigated the effect of these alleles on DNA damage response. Genetic experiments with strains bearing the mutant alleles revealed that mutagenic translesion synthesis, nucleotide excision repair, and homologous recombination are all regulated through residues at the subunit interface. Moreover, genetic characterization of one of our mutants identifies a new sub-branch of nucleotide excision repair. Based on these results we conclude that residues at the subunit boundary of PCNA are not only important for the formation of the trimer structure of PCNA, but they constitute a regulatory protein domain that mediates different DNA damage response pathways, as well.

## Introduction

PCNA was first discovered as a cell-cycle dependent antigene in human cells [1], and later identified as the processivity factor of the replicative DNA polymerases of eukaryotes [2–5]. It forms a ring-shaped structure that encircles DNA and can freely slide along it together with the replicative polymerase attached to it through direct protein-protein interactions [6, 7]. This way PCNA tethers the replicative polymerase to DNA and prevents its dissociation during DNA synthesis. Besides the replicative polymerases, Polε and Polδ, PCNA interacts with other members of the replisome as well [8–11], and it plays a key role in coordinating the steps of lagging strand synthesis [12].

In addition to its essential role in replication, the involvement of yeast PCNA in DNA damage response has been indicated by the sensitivity of many PCNA mutants to DNA damaging agents [13, 14]. The role of PCNA in DNA repair processes could be explained solely by its involvement in the synthesis step as an accessory factor for replicative polymerases. During repair the DNA lesion is excised from one DNA strand leaving a single-stranded gap behind. Repair synthesis, carried out by replicative polymerases attached to PCNA, fills the gap and ligation seals the nick. However, yeast PCNA also shows interaction with a number of repair factors acting outside of the synthetic step. It interacts with base excision repair proteins: the uracil DNA glycosylase Ung1 [15], and the abasic site endonuclease Apn2 [16]. It binds the nucleotide excision repair (NER) endonuclease Rad2 [17]. It also interacts with the mismatch repair proteins Msh2, Msh3, Msh6, and Mlh1 [18–20]. Recently, a direct interaction was shown between yeast PCNA and Rad54, a protein with several functions in homologous recombination (HR) [21, 22]. These diversified connections suggest that PCNA has additional roles in the repair processes; it might help localize the repair factors to damage sites, or it could coordinate the repair steps.

A key coordinating role of yeast PCNA has been well established in DNA damage tolerance, where different mechanisms enable damage-stalled replication forks to resume synthesis in the presence of damage. Through its post-translational modifications PCNA controls which tolerance pathway becomes active. Sumoylation of its lysine 164 residue inhibits the Rad52 governed recombination pathway by binding the anti-recombinase Srs2 that dismantles Rad51 nucleoprotein filaments [23–25]. Ubiquitylation of the same residue, on the other hand, activates the Rad6-dependent damage tolerance pathway [26–28], where the first step is the monoubiquitylation of PCNA by the Rad6- Rad18 ubiquitin conjugase/ligase complex [29]. Monoubiquitylated PCNA (mUB-PCNA) activates translesion synthesis (TLS), where specialized, so-called TLS DNA polymerases take over synthesis from the replicative polymerase and bypass the lesion [30]. TLS polymerases have a PCNA-binding motif, and an additional ubiquitin-binding motif as well, that enhances their affinity toward mUB-PCNA [31–34]. The active sites of TLS polymerases are non-restrictive enabling them to synthesize through several different lesions [35–40]. As a result, they frequently introduce errors during bypass leading to increased mutagenesis. However, assembling a polyubiquitin chain on the already monoubiquitylated residue of PCNA by Rad5/Mms2/Ubc13 activates transient template switching, during which the undamaged newly synthesized daughter strand serves as template resulting in error-free damage bypass [41]. In the polyubiquitylation step Rad5 is the ubiquitin ligase, and Mms2 together with Ubc13 acts as an ubiquitin conjugase [42]. PCNA physically interacts with the ubiquitin ligases Rad18 and Rad5, and also with the TLS DNA polymerases Rev1 and Polη [26, 31, 43].

The PCNA ring is a homotrimer with the monomers in a head to tail arrangement [7]. Each monomer consists of two domains, the N-terminal, and C-terminal domains, and the interdomain connecting loop (IDCL) bridging the two domains together. The IDCL and the C-terminal part contain the binding surfaces for the majority of the PCNA interacting partners identified so far in yeast. Most PCNA interacting proteins like Polδ, Polη, and Rad2 possess a conserved PCNA interacting protein (PIP) motif that mediates interaction with PCNA by binding the hydrophobic pocket of IDCL [17, 31, 44]. Disruption of the PIP motif, or mutations in the IDCL that abolish the interaction, inhibits the function of these proteins. In some cases though, as it has been shown for Fen1 and Apn2, interaction in the absence of DNA is mediated by the IDCL, but functional interaction on DNA is dependent upon the C-terminal region of PCNA [16, 45]. Moreover, in other cases as with Cdc9, both the IDCL and the C-terminal region are required to form a complex with PCNA in the absence, and also on DNA [11]. The C-terminal part has been shown to be the docking site for Polε, as well [44].

In this study we investigated the role of the subunit interface of yeast PCNA in DNA damage response. Through the genetic characterization of point mutant PCNA alleles we show that residues at the subunit interface mediate several DNA damage response pathways like translesion DNA synthesis, nucleotide excision repair, and homologous recombination. Also, with the help of a PCNA mutant we identify a new sub-branch of nucleotide excision repair. These results reveal a new, regulatory function of the PCNA subunit interface.

## Materials and Methods

### Yeast strains and plasmids

Yeast strains used for the genetic studies derived from BY4741 (*MATa*, *his3*-Δ*1*, *leu2*, *met15*, *ura3*) (Euroscarf) and EMY74.7 (*MAT*a, *his3*-Δ*1*, *leu2-3*, -*112*, *trp1*Δ, *ura3-52*). To engineer *pol30* yeast strains, first the strains were transformed with a plasmid expressing the wild type (wt) *POL30* gene from a 2 kilobase (kb) genomic MluI fragment spanning from -185 to 1070 nucleotide (nt) after the STOP codon (pID908 YCplac111 backbone). The strains were kept in selective media to maintain the plasmid. Next the endogenous *POL30* gene was deleted from -185 to 650 nt by gene replacement. After that the plasmid served as the sole source of PCNA. Additional gene deletions were generated in the *pol30* strains containing the wt (pID908), or a mutant PCNA expressing plasmid by the gene replacement method. The wild type PCNA expressing plasmid was replaced by a plasmid expressing a mutant PCNA using plasmid shuffling. To generate mutant PCNAs, first site specific point mutations were created in a copy of the *POL30* gene cloned into pUC19 (pID2) by a PCR based method according to the Stratagene “Quick Change Site Directed Mutagenesis” protocol. The mutations were confirmed by sequencing, and the 2 kb MluI fragment of *POL30* bearing the appropriate mutations were cloned into the YCplac111 yeast vector resulting in pID70 (II99,100AA PCNA), pID87 (D109A PCNA), pID56 (II181,182AA PCNA), and pID40 (Y114A PCNA). Unless specified, all strains were grown at 30°C. The protease deficient yeast strain BJ5464 (*MATα*, *his3*-Δ*200*, *leu2*-Δ*1*, *trp1*Δ, *ura3-52*, *pep4*::*HIS3*, *prb1*-Δ*1*.*6R*, *can1*) (ATCC stock centre) was used for over-expression of proteins used in glutathione S-transferase (GST) pull-down experiments. For purification, proteins were expressed in yeast in N-terminal fusion with GST from plasmids pID207 (wt PCNA), pID537 (II99,100AA PCNA), pID871 (II181,182AA PCNA), pID856 (Rad54), pID864 (Rev1-PAD), and pID460 (Rev1) under the control of a galactose inducible phosphoglycerate promoter (pBJ842 backbone) [46].

### Sensitivity and growth assays

In all sensitivity assays non-selective media was used, because yeast cells bearing a PCNA expressing plasmid as the sole source of PCNA could be maintained without selection, as the loss of the plasmid results in cell death due to the essential functions of PCNA. For qualitative sensitivity assays a single colony from each strain was inoculated into YPD (yeast extract-peptone-dextrose) media and grown overnight. From these starter cultures cells were serial diluted in ten fold dilution steps, spotted onto plates and the desired treatments were applied. For ultraviolet light (UV) assays plates were irradiated with the indicated UV doses and incubated in the dark at 30°C for 2–3 days. For all other sensitivity assays ten fold serial dilutions of the given strains were spotted on plates and incubated at 30°C for 2–3 days. Methyl methanesufonate (MMS) and hydroxy-urea (HU) sensitivities were assayed on YPD plates supplemented with the indicated amount of the chemicals (Sigma-Aldrich). X-ray sensitivity was also examined on YPD plates exposed to the indicated dose of radiation (Trakis XR-11). Bleomycin sensitivity was assayed on synthetic complete (SC) medium supplemented with the specified amount of bleomycin-sulfate (Sigma-Aldrich). In all qualitative spot assays several doses of the given agents were applied, but only plates with the most appropriate doses are presented in figures.

For quantitative analysis of UV sensitivity cells were spread onto YPD plates at appropriate dilutions and irradiated with UV light (254nm) for varying times to apply the specified dosage. Plates were incubated in the dark at 30°C and colonies were counted after 3–4 days. Viability was calculated as the ratio of the number of colonies on UV-treated plates versus the colony number on the corresponding non-irradiated plate. MMS induced killing was quantitated by first harvesting 5 X 10^7^ cells from overnight cultures, resuspending them in 1 ml 50 mM KPO_4_ buffer pH 7.0 containing the desired concentration of MMS, and incubating the cells with shaking at 30°C. After 20 minutes incubation, 1 ml of 10% sodium thiosulfate was added to the cultures to inactivate MMS. Viability was determined by plating the appropriate dilutions of the cultures onto YPD plates and counting the colonies after 3–4 days incubation at 30°C. Viability was calculated as the ratio of the number of colonies on MMS containing plates versus the colony number on the corresponding non-MMS plate.

Growth rates were compared by plating ~100 cells onto YPD plates, incubating them at the indicated temperatures for 2 days. The efficiency of cell cycle progress was further evaluated by checking cellular morphology at low temperature. Briefly, cells were first grown at 30°C to 1 X 10^6^/ml in YPD and then at 14°C for 12.5 hours. Cells were fixed in 3,7% formaldehyde for 3 hours and washed in 0.1 M K_3_PO_4_ solution, sonicated and observed under the microscope. ~ 300 cells were counted for each strain, and the percentage of the large budded (dumbbell) cells, representing cells arrested in the cell cycle after the bulk of DNA replication is completed, was determined. Dumbbell cells refer to cells where the sizes of the bud and the mother cell are approximately equal (14).

### Recovery of transcription

Cells were transformed with a pRS426 based yeast 2 micron plasmid (pID835) expressing LacZ under the control of the *GAL1* promoter. Single colonies were inoculated three days prior to the experiment into 1 ml of SC medium containing 2% lactic acid, 3% glycerol, and lacking uracil and glucose. These starter cultures were used to inoculate into fresh 10 ml of the same media to an A_600_:0.1. At A_600_:0.7 each culture was divided into 5 ml aliquots and treated with UV dose of 130 J/m², except for an untreated sample. The cultures were then resuspended in 5 ml of the same media supplemented with 2% galactose. After induction cultures were incubated in the dark, and 1 ml samples were taken at the indicated time points. Cells were washed in Z-buffer (60 mM Na_2_HPO_4_, 39,8 mM NaH_2_PO_4۰_H_2_O, 10 mM KCl, 0,9 mM MgSO_4_, pH 7.0) and lysed in 100 μl of Z-buffer containing 50 mM β-mercaptoethanol using 3 freeze-thaw cycles. Standard o-nitrophenyl β-D—galactopyranoside (ONPG) liquid assay for determination of β-galactosidase activity was performed as described in the CLONTECH Yeast Protocols Handbook (PT2024-1, chapter VI). Percentage of transcription recovery after UV treatment was determined as the ratio of the enzyme activity of UV treated versus the corresponding untreated sample. Presented results were normalised to the transcription recovery rate of the wild type strain that was set to 1.

### UV-induced and spontaneous mutation rates

UV-induced forward mutation frequencies at the *CAN1* locus were measured by comparing the numbers of *can1*^*R*^ colonies at given UV doses, selected on SC medium without arginine and containing canavanine, with the number of survivors on SC medium exposed to the same UV doses.

For measuring spontaneous forward mutation frequencies at the *CAN1* locus, ten single colonies for each strain were picked and diluted in water. Approximately 10 cells from each colony were inoculated into 500 μl YPD in a microtiter plate and grown for 3 days at 30°C. The cultures were plated onto canavanine-containing plates, and the average number of mutants in case of each strain was counted for 10^7^ plated cells. Mutation frequencies were determined using a chart based on the Lea-Coulson fluctuation model.

### Statistical analysis

Statistical analysis was done by the SigmaPlot program (version 12.5 Systat Software, San Jose, CA). Student’s t-test using Excel (Microsoft, Redmond, WA, USA) was applied to compare separate groups. P-values of <0.05 were considered statistically significant. * p<0.05, ** p<0.01, *** p<0.001

### Protein purification and GST-pulldown assays

For purification, PCNA variants, Rad54, and the Rev1-PAD domain were overexpressed in N-terminal fusion with GST and purified on glutathione—Sepharose 4B beads in a buffer containing 50 mM Tris/HCl pH 7.0, 50 mM KCl, 100 mM NaCl, 10% sucrose, 0.5 mM EDTA, 10 mM 2-mercaptoethanol, and protease inhibitors. GST-Rev1 was purified using the same buffer supplemented with 0.1% Triton X-100 and containing 1 M NaCl. In case of the wild type and mutant PCNA proteins, the GST-tag was removed by PreScission protease cleavage in the elution step of the purification. For checking protein complex formation, GST—Rev1-PAD, GST-Rev1, or GST-Rad54 (5–5 and 3 μg, respectively) immobilized on glutathione—Sepharose beads was incubated with purified PCNA variants (3–5 μg) overnight on ice in buffer containing 50 mM Tris/HCl, pH 7.5, 100 mM NaCl, 1 mM EDTA, 1 mM DTT, 10% glycerol, 0.01% Nonidet P-40. Beads were washed five times with the same buffer and bound proteins were eluted in the same buffer either containing 20 mM reduced glutathione (for Rad54 and Rev1-PAD) or PreScission protease (for Rev1). Various fractions were analyzed by SDS/PAGE.

## Results

### Replication and DNA damage response in the PCNA mutants

**Fig 1:**
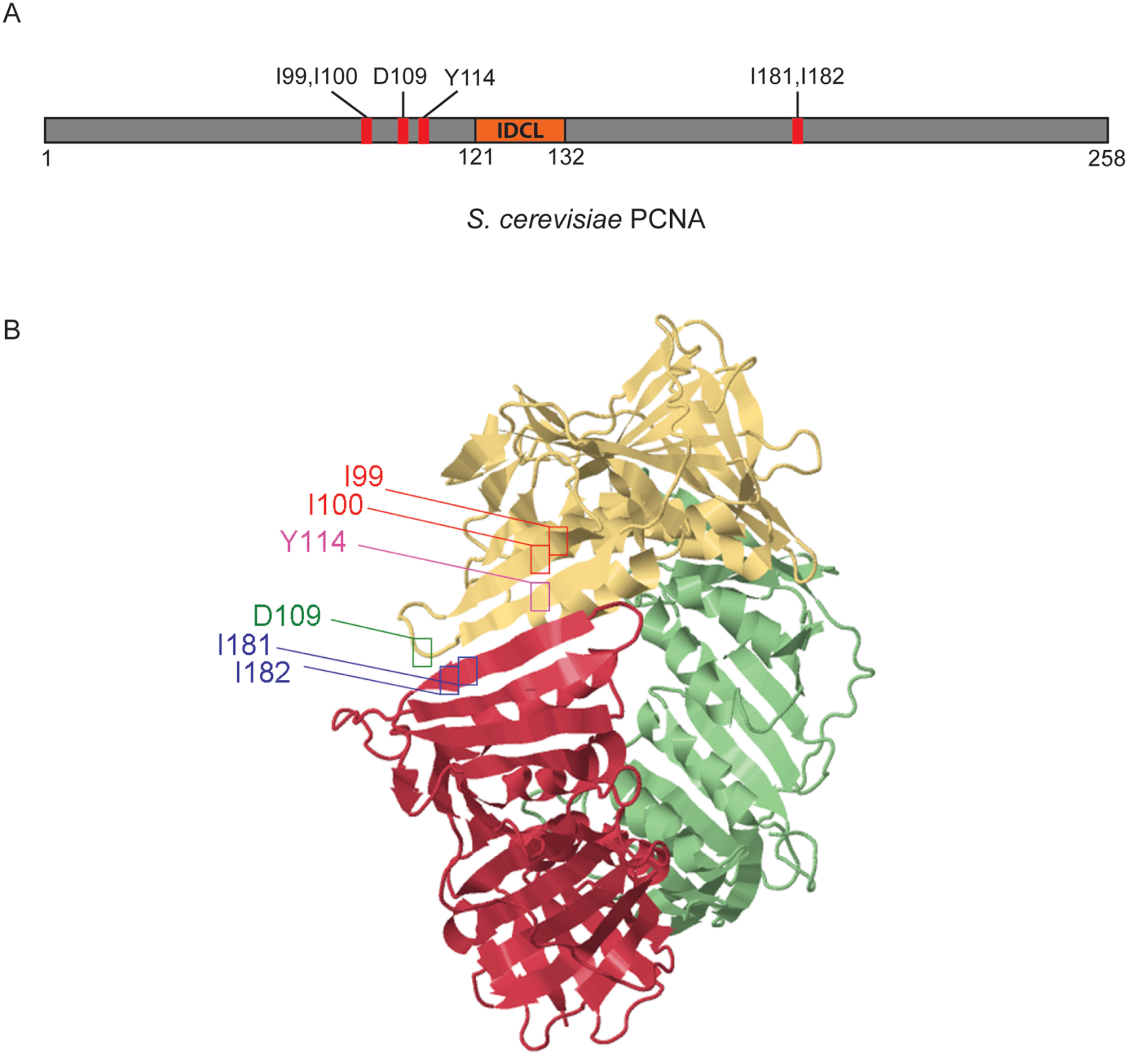
The positions of the mutated amino acids in the structure of PCNA. (A) Bar diagram of yeast PCNA. The positions of the altered amino acids, and of the IDCL are indicated. (B) The positions of the mutated amino acids at the subunit interface are shown in different colors in a schematic ribbon diagram representing the three dimensional structure of the PCNA trimer (source: Krishna, T.S.R at al. from RCSB PDB, PDB ID:1PLR) [7]. The monomers are depicted in different colors.

In order to investigate the involvement of different parts of PCNA in DNA damage response, we generated several mutant alleles with the alterations spreading throughout the protein sequence of PCNA. In all mutants the original amino acids were replaced by alanine. We chose alanine for substitution because its side chain consists of a single methyl group. Due to that alanine can be regarded as an equivalent to other amino acids truncated back to the first atom in their side chain. This way the contributions of the side chains of the different amino acids to the function of PCNA can be determined. In this study we present the characterization of mutations situated at the subunit interface. The positions of the mutated amino acids are shown in Fig 1. The isoleucine replacement mutations (II99,100AA) and the mutated tyrosine (Y114A) are in close vicinity of each other, on two parallel β-strands of the same subunit. The aspartic acid change (D109A) and mutations at double isoleucines (II181,182AA) are spatially adjacent, but they are on different subunits. The mutant alleles were expressed under the PCNA promoter from yeast centromeric plasmids in yeast cells lacking the genomic copy of the *POL30* gene encoding for PCNA.

**Fig 2:**
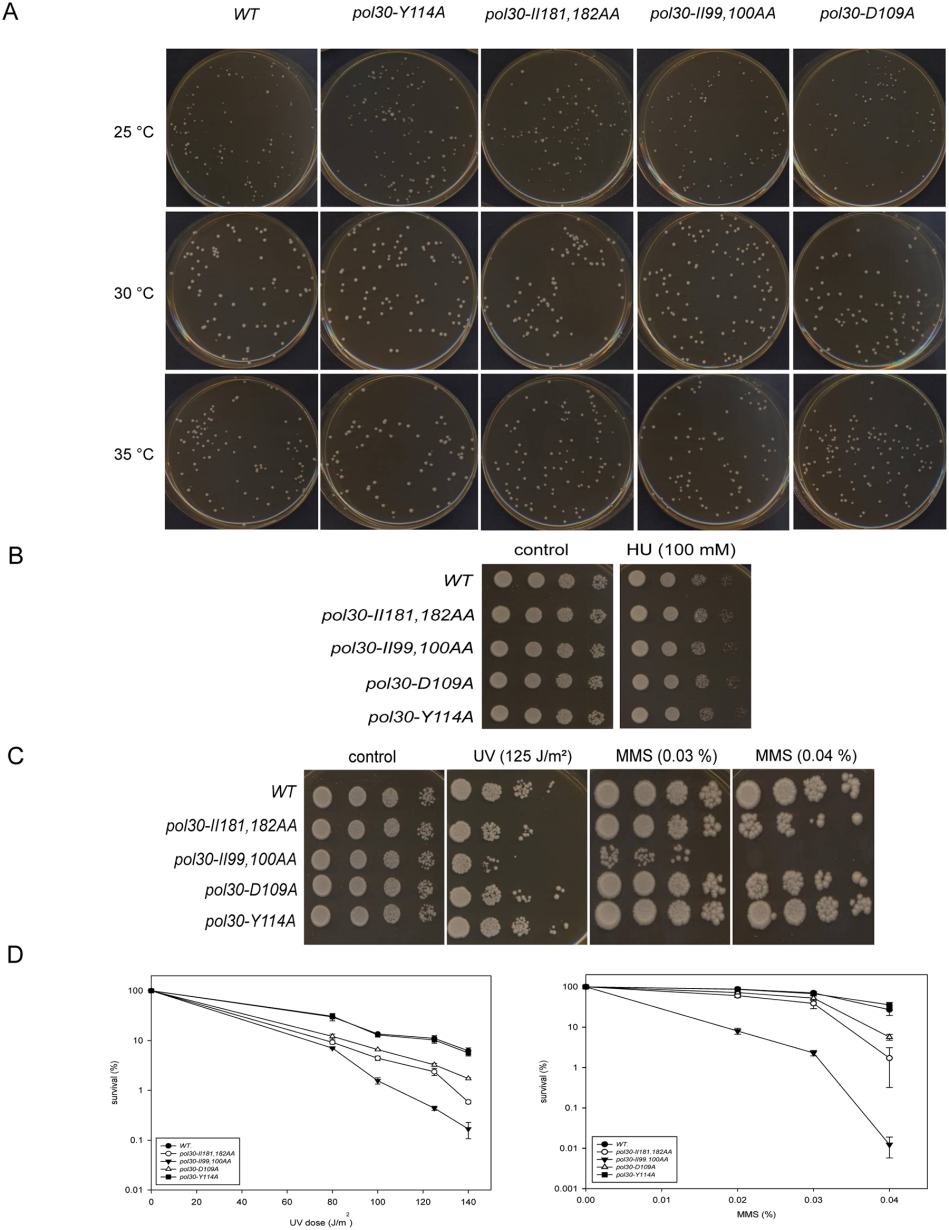
Phenotypic characterization of the mutants. (A) Growth of the strains at different temperatures. Approximately 100 cells of each strain were plated on rich medium and the plates were incubated at the indicated temperatures for 2 days. (B) Sensitivity of the strains to HU. Ten fold serial dilutions of the indicated strains were spotted on YPD plates containing the given amount of HU. (C) Sensitivity of the strains to DNA damaging agents. Cells were spotted as described for (B) on plates either exposed to the indicated UV dose, or containing the indicated amount of MMS. For (B) and (C) several HU, UV, and MMS doses were applied, but only plates with the most appropriate doses are presented. (D) Quantitative assays of UV and MMS induced killing of the indicated strains. The results represent the average of three experiments. Standard deviations are indicated.

We were looking for mutations that affected the DNA damage response function of PCNA, but did not interfere with its essential role in replication. Temperature sensitive growth, and sensitivity to the replication inhibitor hydroxy-urea (HU) are indicative of perturbation in replication. To exclude mutations that affected normal replication, first we checked the growth rate of yeast strains, bearing the mutant alleles, at different temperatures, and also their sensitivity to HU. As Fig 2A shows, none of the strains grew slower at either elevated or at lower temperature, nor did they show the large budded phenotype at 14°C (Table 1), previously observed with PCNA mutants with replication defects [13, 14]. Also, they exhibited wild type sensitivity to even high concentration of HU (Fig 2B). These data indicated that the mutations did not disrupt the *in vivo* conformation of PCNA, or its interactions necessary for normal replication.

**Table 1:** Percentage of cells displaying the large budded (dumbbell) morphology of yeast strains expressing the PCNA variants.

Genotype	% of dumbbell cells
*WT*	6.7 ± 1.0
*pol30-II181*,*182AA*	6.8 ± 1.1
*pol30-II99*,*100AA*	8.1 ± 0.7
*pol30-D109A*	7.7 ± 1.4
*pol30-Y114A*	7.2 ± 1.6

Cells were grown at 30°C then at 14°C for 12.5 hours, and after washing and fixation ~300 cells of each strain were examined for cell morphology. Cells displaying the large budded phenotype (dumbbell cells), where the sizes of the mother cell and the bud are approximately equal indicative of replication defect, were counted. The results represent the average of three experiments. Standard deviations are indicated.

Next we checked the sensitivity of the mutant strains to DNA damage using ultraviolet light (UV) treatment, or the methylating agent methyl-methanesulfonate (MMS). As it is shown in Fig 2C and 2D, three mutants exhibited varying mild sensitivity to both UV and MMS at high doses, but the Y114A mutant behaved like the wild type strain. Because of its insensitivity, this mutant was excluded from further investigation.

### The II99,100AA mutations in PCNA inhibit mutagenesis

**Fig 3:**
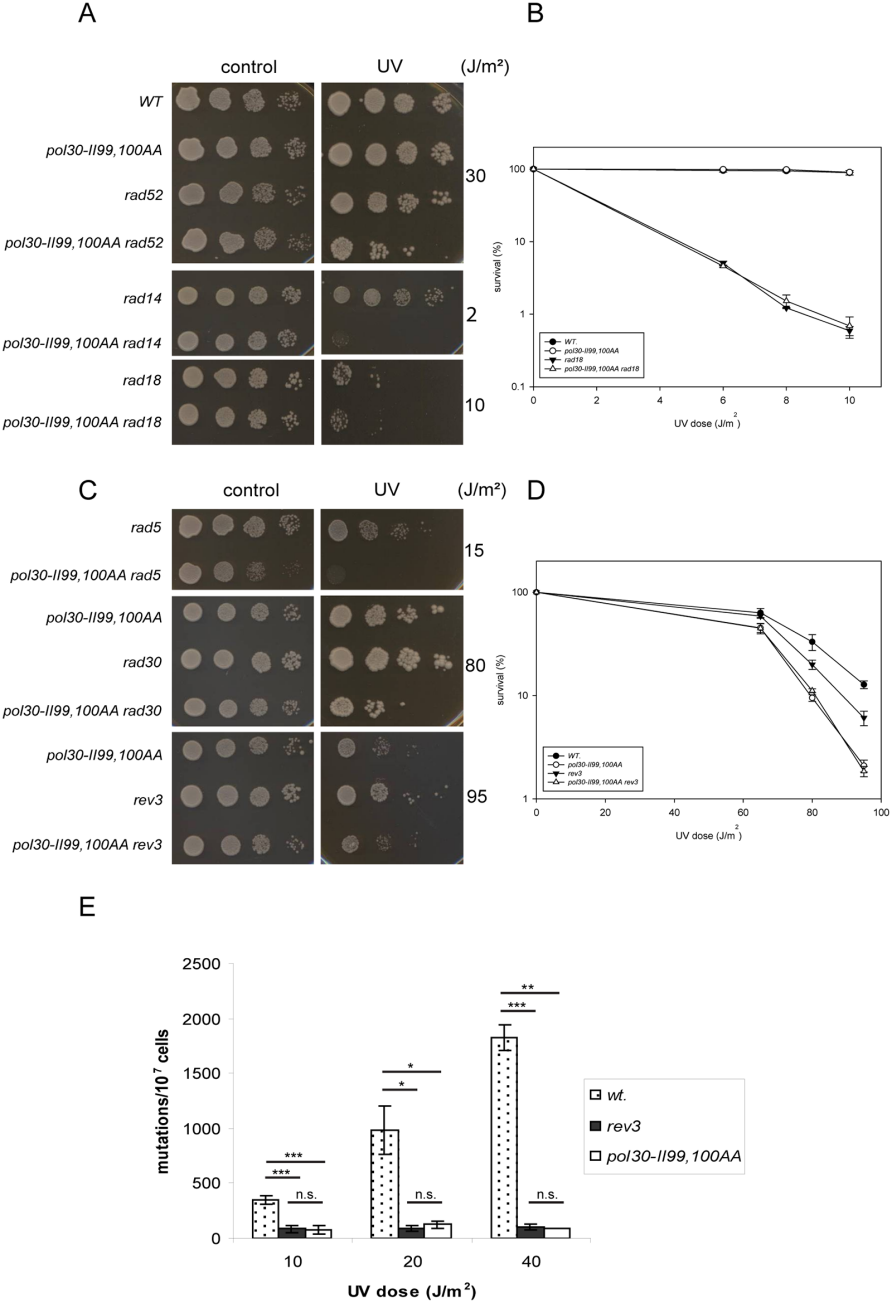
In the II99,100AA PCNA mutant strain the *REV3* branch of translesion synthesis is inactivated. (A-B) The II99,100AA PCNA mutant shows epistasis with *RAD18* upon UV treatment. (C-D) The II99,100AA PCNA mutant affects the Rev3 pathway. On (A) and (C) ten fold serial dilutions of cells with the indicated genotypes were spotted on plates and exposed to UV doses indicated on the right. (B) and (D) show quantitative analysis of UV-survival of the indicated strains. (E) UV-induced mutagenesis is abolished in the II99,100AA PCNA mutant strain. Forward mutation rates at the *CAN1* locus were determined after exposing the cells to the indicated UV doses. (B), (D), and (E) represent the average of three experiments. Standard deviations are indicated. P-values representing the significance of difference are also shown on (E). *:p<0.05 ** p<0.01, *** p<0.001 ns: no statistical difference.

In order to determine which DNA damage response pathway was affected in the PCNA mutant strains, we performed yeast epistasis analysis. We generated double mutants by deleting the genes of representative members of the main DNA repair and damage tolerance pathways in the PCNA mutants, and examined their sensitivity to UV. In the II99,100AA PCNA mutant strain deletion of either *RAD14*, representing NER, or *RAD52*, representing HR, resulted in much higher sensitivity compared to the single mutants suggesting that NER and HR were functional (Fig 3A). Contrary to that, the II99,100AA PCNA mutant showed epistasis with *RAD18*, since the *rad18 pol30-II99*, *100AA* strain exhibited the same UV-sensitivity as the *rad18* strain (Fig 3A and 3B). This suggested that the II99,100AA PCNA mutations affected the *RAD6/RAD18* governed DNA damage tolerance.

The *RAD6/RAD18* pathway can be divided into the *RAD5*, the *RAD30*, and the *REV3* branches based on UV epistasis analysis [47]. To investigate which branch was affected by the mutations, we deleted the above genes in the II99,100AA PCNA mutant strain. Additional deletion of either *RAD5* or *RAD30* caused increased sensitivity, but deletion of *REV3* in the *pol30-II99*, *100AA* strain did not sensitized the strain further (Fig 3C and 3D). These data suggested that the II99,100AA PCNA mutations affected the mutagenic *REV3* branch of the *RAD6/RAD18* pathway.

The *REV3* branch is responsible for most of the induced mutations, and it also contributes to spontaneous mutagenesis. This branch consists of the TLS polymerases Rev1, and Polζ formed by Rev3 and Rev7, and also of the Def1 protein promoting the degradation of the catalytic subunit of the replicative polymerase Polδ for induced mutagenesis [48]. The lack of any of these proteins lowers spontaneous mutagenesis and inhibits induced mutagenesis. To confirm that the II99,100AA PCNA mutations affected the *REV3* branch, we checked spontaneous and induced mutagenesis in the II99,100AA PCNA mutant strain. Indeed, both induced and spontaneous mutagenesis were decreased in the II99,100AA PCNA strain to a level similar to that of the *rev3* strain (Fig 3E and Table 2). These results confirmed that the error-prone *REV3* branch was inactive in the II99,100AA PCNA mutant strain.

**Table 2:** Spontaneous mutation rates in yeast strains expressing the PCNA variants.

Genotype	Spontaneous mutation rates
*WT*	9.63×10^-7^ ± 1.08×10^-7^
*pol30-II99*,*100AA*	6.92×10^-7^ ± 0.75×10^-7^
*pol30-D109A*	9.57×10^-7^ ± 0.83×10^-7^
*pol30-II181*,*182AA*	9.23×10^-7^ ± 0.65×10^-7^
*rev3Δ*	6.44×10^-7^ ± 0.51×10^-7^

Spontaneous forward mutation rates at the *CAN1* locus were determined by fluctuation analysis using ten independent cultures for each strain. The results represent the average of three experiments. Standard deviations are indicated.

### The II99,100AA mutations impede the PCNA-Rev1 interaction

**Fig 4:**
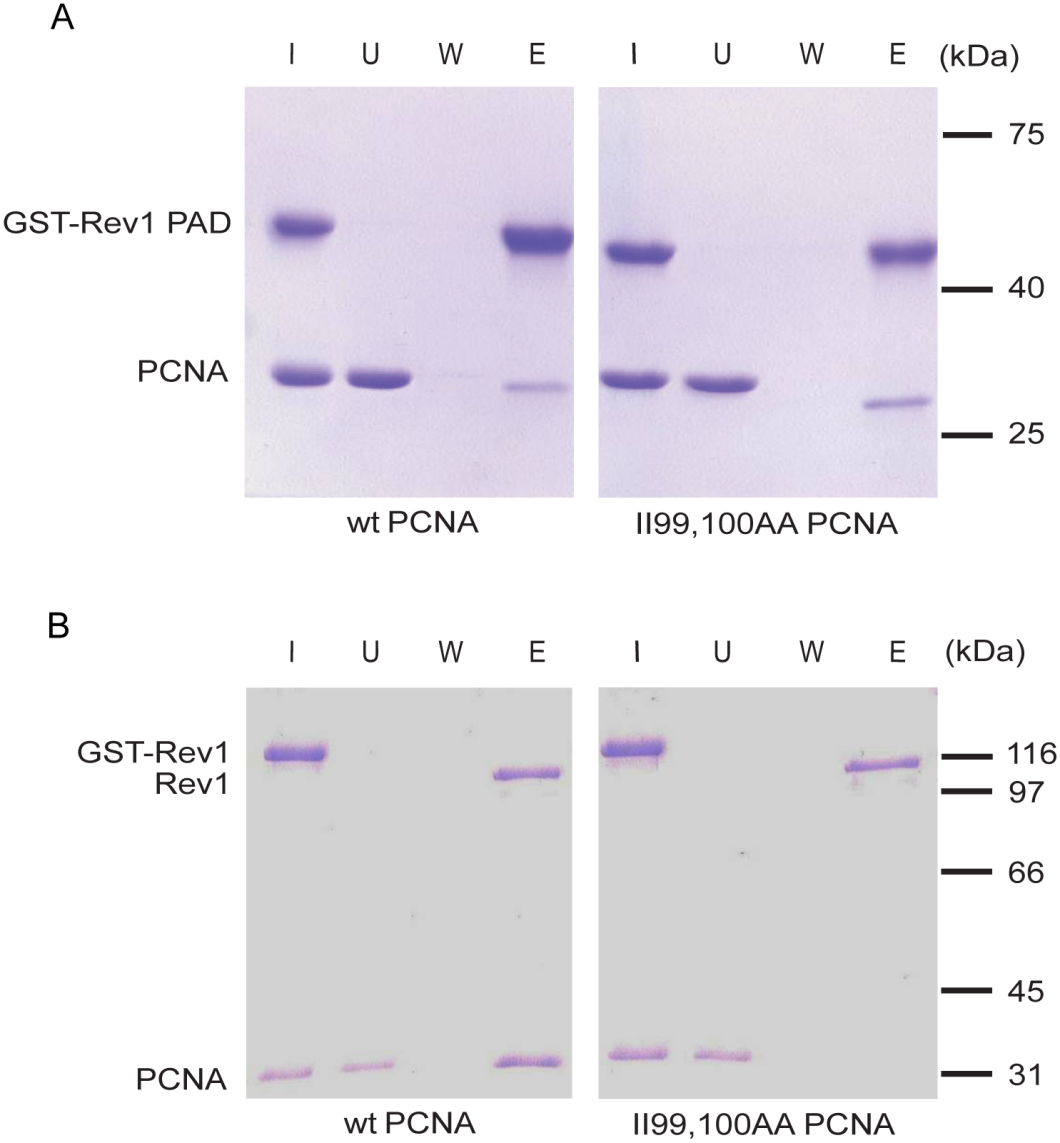
Complex formation between PCNA and Rev1. (A) GST-pull-down assay with Rev1-PAD and wild type, or II99,100AA PCNA. GST-Rev1-PAD (5 μg) immobilized on glutathione-Sepharose beads was incubated with purified wild type (left panel), or II99,100AA mutant PCNA (right panel) (5–5 μg). After washing bound proteins were eluted with glutathione. Aliquots of each sample, taken from the input (I), from the unbound fraction (U), from the last wash (W), and from the glutathione-eluted proteins (E), were analyzed on 10% SDS polyacrylamide gel. (B) GST-pull-down with Rev1 (5 μg) and wild type, or II99,100AA mutant PCNA (3–3 μg). Experiments were carried out as described for (A), but GST-Rev1 was used instead of GST-Rev1-PAD, and elution was achieved by PreScission protease cleavage of Rev1 from GST. The position of the cleaved Rev1 without the GST tag in the elution fraction is also marked.

The E113 residue of PCNA was previously shown to mediate interaction between PCNA and the polymerase-associated domain (PAD) of Rev1 [43]. Replacement of this residue by glycine abolished the interaction, and inhibited mutagenesis like the *REV3* deletion indicating that the Rev1-PCNA interaction was essential for translesion synthesis by the mutagenic branch. Since the mutated isoleucine amino acids in our mutant are spatially close to E113, we investigated whether the II99,100 residues of PCNA were also involved in mediating the interaction with Rev1-PAD. For this purpose, we performed pull-down experiments using untagged purified wild type, or the II99,100AA mutant PCNA protein together with a truncated Rev1 consisting of amino acids 623 to 767 that included the PAD region. Rev1-PAD was N-terminally fused to glutathione S-transferase (GST). First we immobilized GST-Rev1-PAD on glutathione-sepharose beads, and then incubated it with the wild type, or the II99,100AA mutant PCNA. After washing bound proteins were released from the beads by addition of glutathione. As shown in Fig 4A, GST-Rev1-PAD and wild type PCNA co-eluted from the column indicating an interaction between the two proteins, in good agreement with the published results. However, GST-Rev1-PAD also interacted with the II99,100AA mutant PCNA protein suggesting that the II99,100AA mutations did not disrupt the binding of Rev1-PAD. The II99,100AA mutant PCNA behaved like the wild type even when repeating the experiment with varying (50–150 mM) salt concentrations (data not shown).

To fortify these results, we repeated the experiments using purified full-length Rev1 instead of Rev1-PAD. In these experiments we could detect Rev1 in the elution fraction with wild type PCNA, but not in the elution with the II99,100AA mutant PCNA (Fig 4B). These data indicated that even though full-length Rev1 interacted with wild type PCNA, it could not interact with the II99,100AA mutant. Taken together our data revealed that the II99,100 residues of PCNA were required for establishing the interaction between PCNA and Rev1.

### The D109A PCNA mutant unmasks a new sub-pathway of nucleotide excision repair

**Fig 5:**
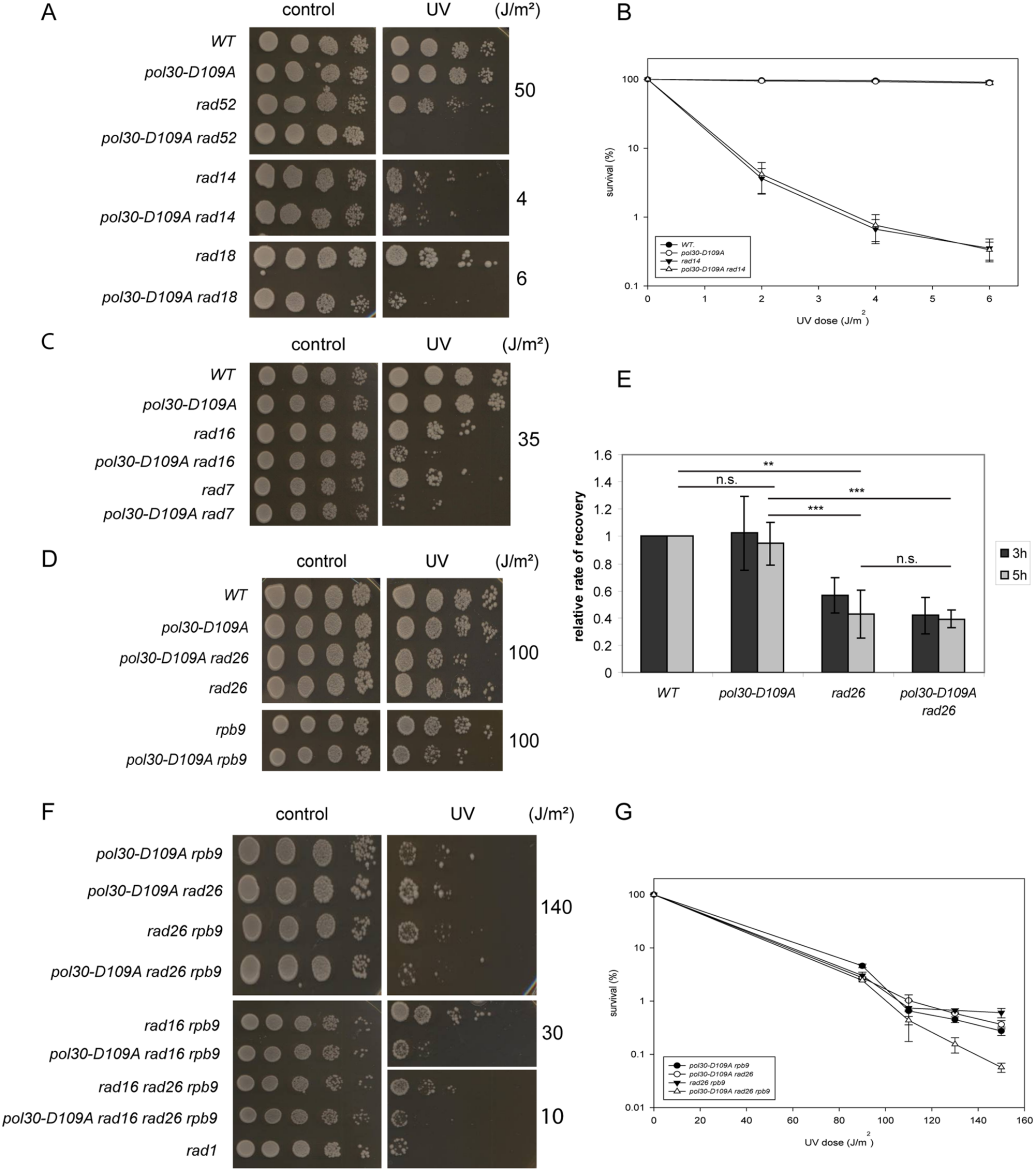
A new sub-branch of NER is inactivated in the D109A PCNA mutant. (A-B) The D109A PCNA mutation is epistatic with *RAD14*. (C) GGR mutants *rad7* and *rad16*, and (D) TC-NER mutants *rad26* and *rpb9* are further sensitized by the D109A PCNA mutant. (E) Transcription of the *LacZ* gene recovers in the D109A PCNA mutant as fast as in the wild type. For transcriptional recovery, β-galactosidase activity was measured at the indicated time points after UV-treatment of cells followed by galactose induction of the *LacZ* gene. Recovery was determined as the ratio of the enzyme activity of UV treated and the corresponding untreated samples. The results were normalised to the recovery rate of the wild type strain that was set to 1. (F-G) The D109A PCNA mutant further sensitizes strains with combinations of NER mutations. In panels (A), (C), (D), and (F) ten fold serial dilutions of strains with the indicated genotypes were spotted on YPD plates and irradiated with the indicated UV doses. Panels (B) and (G) show quantitative UV survival of strains. In panels (B), (E), and (G) the results represent the average of three experiments. P-values representing the significance of difference are also shown on (E). *:p<0.05 ** p<0.01, *** p<0.001, ns: no statistical difference.

To assign the D109A mutant PCNA to a DNA damage response pathway, we performed similar genetic analysis as with the previous mutant. Our results showed that the D109A PCNA mutation strongly sensitized both the *rad52* and the *rad18* strains, but it showed epistasis with *RAD14* suggesting that the D109A PCNA mutation affected NER (Fig 5A and 5B). NER can be divided into two branches, global genome repair (GGR) responsible for the repair of DNA damage in non-transcribed regions of the genome and in the non-transcribed strand of active genes, and transcription-coupled NER (TC-NER) acting on the transcribed strand of active genes [49, 50]. TC-NER can be further divided into two sub-pathways, the *RAD26* and the *RPB9* dependent pathways [51, 52]. In order to determine which sub-pathway was affected by the D109A PCNA mutant, we first examined its relation to the *RAD16* and *RAD7* genes, the products of which form together a complex involved in the DNA damage recognition and also in post-incision steps of GGR [53–55]. As it is shown in Fig 5C, the D109A PCNA mutation strongly increased the UV-sensitivity of both the *rad16* and the *rad7* strains indicating that GGR was functional in the D109A PCNA mutant strain.

Next we investigated the relation of the D109A PCNA mutant to *RPB9* and *RAD26* functioning in the two sub-pathways of TC-NER. The D109A PCNA mutation was not epistatic with *RPB9*, since a small, but consistent increase in UV-sensitivity could be detected in the *pol30-D109A rpb9* double mutant (Fig 5D). A similar degree of increase in sensitivity could be observed, when we deleted *RAD26* in the D109A PCNA mutant strain. These results suggested that both the Rpb9 and Rad26 dependent TC-NER pathways were active in the D109A PCNA mutant strain.

In order to verify that the Rad26 dependent TC-NER was indeed functional, we investigated the recovery of transcription in the D109A PCNA mutant strain after DNA damaging treatment. In the absence of Rad26 transcriptional recovery is delayed in UV-treated cells compared to wild type, because of the inactivation of a TC-NER pathway. As our results show, the induction of the bacterial *LacZ* gene driven from the galactose inducible *GAL1* promoter recovered as fast in the D109A single mutant as in the wild type, and transcriptional recovery was delayed to the same degree in both the *rad26* and the *pol30-D109A rad26* strains (Fig 5E). These experiments confirmed that the D109A PCNA mutation did not compromise the function of the *RAD26* branch.

Based on these results we concluded that a separate yet unknown pathway of NER was inactivated in the D109A PCNA mutant strain. In order to corroborate this hypothesis, we created triple and quadruple mutant strains and investigated their sensitivity to UV irradiation. Indeed, at high UV doses the *pol30-D109A rad26 rpb9* triple mutant was more sensitive than any of the double mutants (Fig 5F and 5G) further supporting that the D109A PCNA mutation did not affect either TC-NER pathways [52]. Moreover, the *pol30-D109A rad16 rad26 rpb9* quadruple mutant exhibited the same hypersensitivity to UV as the completely NER deficient *rad1* strain, both of them being more sensitive than the *rad16 rad26 rpb9* strain (Fig 5F). These results substantiated that the D109A PCNA mutation inactivated a new, separate sub-branch responsible for the residual NER activity in the *rad16 rad26 rpb9* strain, and resulting in complete blockage of NER in the quadruple mutant.

### The II181,182AA PCNA mutant affects the repair of UV-induced DNA damage by the RAD52 governed homologous recombination

**Fig 6:**
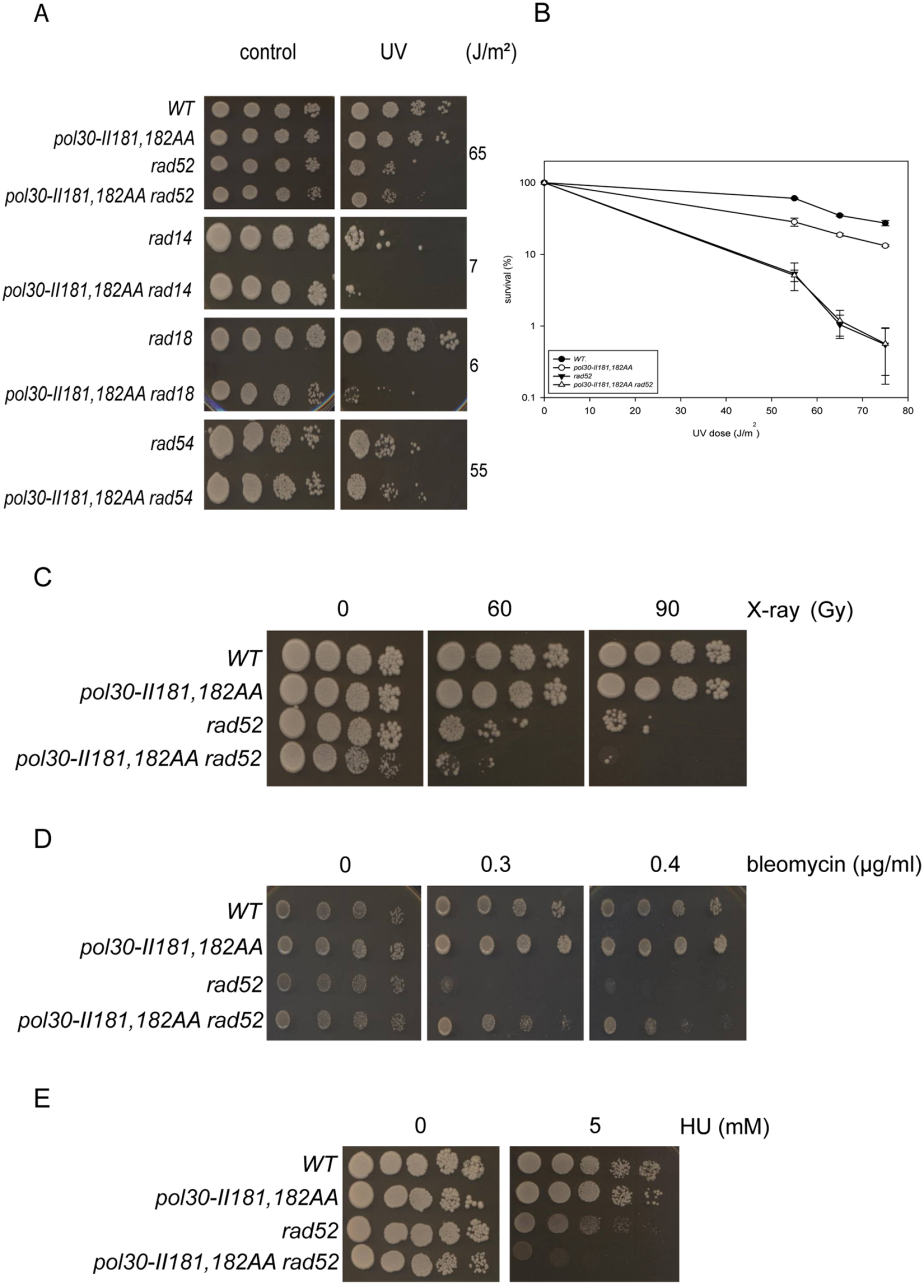
The II181,182AA PCNA mutations affect HR differently based on the nature of the treatment of the cells. (A) The II181,182AA PCNA mutant is epistatic to both *RAD52* and *RAD54* upon UV-treatment. (B) Quantitative analysis of UV-survival of the *rad52 pol30-II181*,*182AA* double mutant. (C) X-ray (D) bleomycin (E) HU sensitivity of the *rad52 pol30-II181*,*182AA* double mutant strain. In panels (A), (C), (D), and (E) ten fold serial dilutions of strains with the indicated genotypes were spotted on YPD plates containing the given amount of bleomycin, or HU, or irradiated with the indicated UV or X-ray doses.

Genetic experiments with the third II181,182AA PCNA mutant revealed the increased sensitivity of the *pol30-II181*, *182AA rad14* and *pol30-II181*,*182AA rad18* double mutant strains to UV, compared to the single mutants (Fig 6A). However, an epistatic relation was observed between the PCNA mutant and *RAD52* functioning in HR (Fig 6A and 6B). These results suggested that the II181,182AA PCNA mutant affected the *RAD52* governed homologous recombination pathway. This was confirmed by detecting an epistatic relationship between the II181,182AA PCNA mutant and *RAD54*, another member of the HR pathway (Fig 6A).

Besides repairing UV damage, the *RAD52* governed HR is the predominant pathway in the repair of DNA double-strand breaks in yeast cells. To investigate whether the II181,182AA PCNA mutant is also involved in DNA double-strand break repair, we examined its sensitivity to X-ray, and also to the radiomimetic agent bleomycin, both treatments inducing the formation of DNA double-strand breaks. Though the II181,182AA PCNA mutant itself did not show sensitivity to either of these agents, it increased the sensitivity of *rad52* to X-ray irradiation (Fig 6C), whereas it partially suppressed the bleomycin sensitivity of *rad52* indicated by the elevated resistance of the *rad52 pol30-II181*,*182AA* strain, compared to *rad52* (Fig 6D). Next we investigated the relation of the II181,182AA PCNA mutant to Rad52 upon HU treatment, as the Rad52 pathway is involved in the restart of HU-stalled replication forks. We found that the *pol30-II181*,*182AA* mutant itself behaved like wild type on HU containing plates, whereas the *rad52 pol30-II181*,*182AA* strain was more sensitive than *rad52* (Fig 6E). Taken together these results strongly suggested that the II181,182 residues of PCNA promoted the *RAD52* governed HR in the repair of UV damages, but in case of DNA double-strand break repair and replication restart they facilitated another repair pathway. The surprising difference between the effects of X-ray and bleomycin could probably be due to the distinct spectra of DNA damages caused by these agents beside double-strand breaks, and supported the conclusion that the II181,182 PCNA mutations affected more than one pathway of DNA repair.

### The II181,182AA PCNA can interact with Rad54 *in vitro*

**Fig 7:**
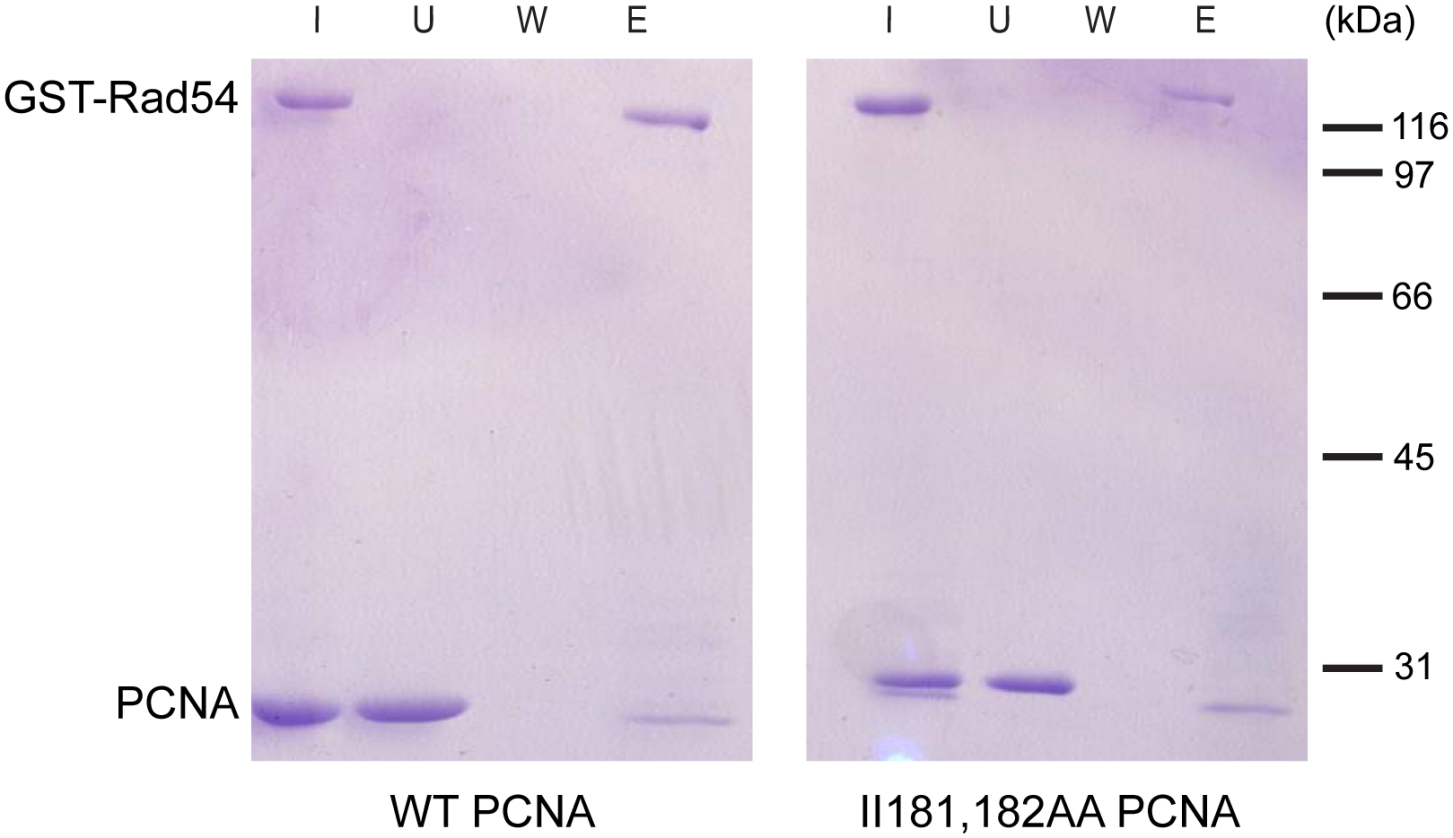
The II181,182AA PCNA behaves like wild type in its interaction with Rad54 *in vitro*. GST-Rad54 (3 μg) bound to glutathione-Sepharose beads was incubated with purified wild type (left panel), or II181,182AA mutant PCNA (right panel) (5–5 μg). After washing bound proteins were eluted with glutathione. Aliquots of each sample, taken from the input (I), from the unbound fraction (U), from the last wash (W), and from the glutathione-eluted proteins (E), were analyzed on 10% SDS polyacrylamide gel.

Among the HR proteins only Rad54 has been shown to interact with PCNA, though the PIP motif identified in Rad54 was shown to be largely dispensable for the interaction [21, 22]. To test, whether the II181,182 residues of PCNA were involved in mediating this interaction, we performed GST pull-down experiments with purified proteins. Our results showed that wild type PCNA and Rad54 co-eluted from the column indicating that the two proteins formed a complex (Fig 7). However, the II181,182AA PCNA protein also co-eluted with Rad54 prompting that the II181,182AA mutations did not disrupt the interaction of PCNA with Rad54 *in vitro*. The II181,182AA mutant PCNA protein behaved like the wild type PCNA even under different reaction conditions, when applying 50–150 mM salt concentrations (data not shown).

## Discussion

In this report, by characterizing three PCNA mutant yeast strains, we found that in addition to its role in maintaining the trimer ring structure, the subunit interface of yeast PCNA also has regulatory functions in several DNA damage response pathways.

In the first strain the II99,100AA PCNA mutations inactivated the Rev3 dependent mutagenic translesion synthesis branch of the Rad6/Rad18 directed DNA damage tolerance. This was demonstrated by epistasis analysis, and by the lack of induced mutagenesis, and lowered spontaneous mutagenesis in the II99,100AA PCNA mutant strain. Another change at the subunit interface of PCNA, described previously in the literature, the E113G mutation also disabled the mutagenic Rev3 branch. The II99,100 amino acids are spatially close to E113, however, our data indicated that the actual roles of these residues differed slightly from each other. Whereas the E113G PCNA mutation prevented the binding of Rev1 by abolishing the interaction between PCNA and the PAD domain of Rev1, the II99,100AA PCNA mutations did not affect the binding of Rev1-PAD to PCNA. Nevertheless, they prevented the binding of the full-length Rev1 to PCNA suggesting that the II99,100 residues of PCNA mediated interaction with Rev1 residues situated outside of the PAD region.

The second strain carried the D109A PCNA mutant that was assigned by genetic means to nucleotide excision repair. Even so, the D109A mutation did not affect any of the known sub-pathways of NER, the GGR or the TC-NER branches, suggesting that a yet unidentified new sub-branch of NER was inactivated by the mutation. This was supported by the increased UV sensitivity of the *pol30-D109A rad16 rad26 rpb9* quadruple mutant compared to the *rad16 rad26 rpb9* triple mutant having all known NER pathways inactivated. Moreover, the quadruple mutant exhibited the same hypersensitivity to UV as the *rad1* strain lacking the endonuclease acting in the damage removal step, and essential for all three known sub-branches of NER. It is intriguing when and how NER could be activated in this new pathway, since it seems independent of TC-NER, and the damage signaling proteins of GGR. We surmise that PCNA itself in complex with another, yet unknown, protein could function as a DNA damage sensor in the new sub-branch of NER. The complex of the two proteins could scan the genome and once they localize DNA damage, they stall and recruit the NER factors for repair to take place. The D109A mutation might hinder the formation of that complex causing the inhibition of the sub-branch. We note though, that the contribution of this pathway to overall NER activity must be minor based on the relative UV-sensitivity of the D109A PCNA strain. Its activity is largely masked by the activity of GGR as its inactivation results in significant UV sensitivity only when GGR is non-functional. This might reflect that the activity of this new sub-branch is restricted to a specific cell cycle stage, or to specific regions of the genome.

In the third strain the II181,182AA PCNA mutations affected the Rad52 governed repair of UV damages. This effect, however, seemed to be limited to the repair of UV damage, as the *rad52 pol30-II181*,*182AA* strain exhibited increased sensitivity to X-ray, and also to the replication inhibitor HU, compared to *rad52*. Taken together these results suggested that the II181,182 residues of PCNA participated in channeling repair into the Rad52 pathway in case of UV-induced DNA damage, but they promoted another pathway in the case of DNA strand break repair, and also in replication fork restart. This another pathway, though, most probably served as a back-up system that became active only in the absence of HR, suggested by the wild type sensitivity of the II181,182AA PCNA mutant itself to X-ray and HU, and by the increased sensitivity of the *rad52 pol30-II181*,*182AA* strain to these agents, compared to *rad52*.

In summary, in this study we identify a new regulatory surface of PCNA. Our findings demonstrate that residues at the subunit interface of PCNA mediate several DNA damage response pathways. Our data, indicating that even spatially close residues can affect different damage response processes, suggest that this part of PCNA, similar to the IDCL, affects the separate pathways via mutually exclusive binding of proteins. As the homotrimer PCNA ring can facilitate the concurrent binding of proteins with overlapping binding sites at separate monomer interfaces, we speculate that PCNA could form one complex with its several partners of the different DNA damage response pathways allowing rapid sampling for the most suitable damage response upon replication stalling. Providing that protein binding at the subunit interface does not interfere with binding at the IDCL, this could occur without major disruption of the replication machinery enabling the cells to react fast to the constantly varying challenges of DNA metabolism. Further analysis of the subunit interface should unravel more details of its diverse regulatory functions.
